# Deciphering the Role of *Klf10* in the Cerebellum

**DOI:** 10.4236/jbise.2022.155014

**Published:** 2022-05-30

**Authors:** Malek Kammoun, Lydie Nadal-Desbarats, Sandra Même, Aude Lafoux, Corinne Huchet, Géraldine Meyer-Dilhet, Julien Courchet, Frédéric Montigny, Frédéric Szeremeta, William Même, Vladimir Veksler, Jérôme Piquereau, Philippe Pouletaut, Malayannan Subramaniam, John R. Hawse, Jean-Marc Constans, Sabine F. Bensamoun

**Affiliations:** 1 Biomechanics and Bioengineering CNRS UMR 7338, Sorbonne University—University of Technology of Compiègne, Compiègne, France;; 2 iBrain CNRS UMR 1253, University of Tours, Tours, France;; 3 Center for Molecular Biophysics, CNRS UPR4301, Orléans, France;; 4 Therassay Platform, University of Nantes, Nantes, France;; 5 INSERM UMR1089, University of Nantes, Nantes, France;; 6 CNRS UMR-5310 and INSERM U-1217, NeuroMyoGène Institute, Villeurbanne, France;; 7 INSERM PST-ASB, University of Tours, Tours, France;; 8 INSERM UMR-S 1180, University of Paris-Saclay, Châtenay-Malabry, France;; 9 Department of Biochemistry and Molecular Biology, Mayo Clinic, Rochester, USA;; 10 CHIMERE EA7516 UPJV, Faire Faces Institute, Amiens, France

**Keywords:** *Klf10*, Cerebellum, Mice, Magnetic Resonance Imaging and Spectroscopy, Metabolomics, Mitochondria

## Abstract

Recent studies have demonstrated a new role for *Klf10*, a Krüppel-like transcription factor, in skeletal muscle, specifically relating to mitochondrial function. Thus, it was of interest to analyze additional tissues that are highly reliant on optimal mitochondrial function such as the cerebellum and to decipher the role of *Klf10* in the functional and structural properties of this brain region. *In vivo* (magnetic resonance imaging and localized spectroscopy, behavior analysis) and *in vitro* (histology, spectroscopy analysis, enzymatic activity) techniques were applied to comprehensively assess the cerebellum of wild type (WT) and *Klf10* knockout (KO) mice. Histology analysis and assessment of locomotion revealed no significant difference in *Klf10* KO mice. Diffusion and texture results obtained using MRI revealed structural changes in KO mice characterized as defects in the organization of axons. These modifications may be explained by differences in the levels of specific metabolites (*myo*-inositol, lactate) within the KO cerebellum. Loss of *Klf10* expression also led to changes in mitochondrial activity as reflected by a significant increase in the activity of citrate synthase, complexes I and IV. In summary, this study has provided evidence that *Klf10* plays an important role in energy production and mitochondrial function in the cerebellum.

## 1. INTRODUCTION

*Klf10*, a Krüppel-like transcription factor that contains three C2 H2 type zinc fingers, was originally discovered in human osteoblasts as a TGF*β* inducible early gene [1]. Following its original discovery, this gene has been shown to play an important role in multiple organs, tissues and cell types. We and others have previously demonstrated that knockout (KO) of this gene in mice (*Klf10* KO) leads to an osteopenic phenotype that occurs only in female animals [2–4]. *Klf10* KO mice also exhibited other phenotypes such as hypertrophic hearts [5], defects in the immune system [6–8], alterations in circadian regulated genes [9] and glucose regulation [10]. Mutations in the *Klf10* gene, and altered expression levels of *Klf10*, have also been observed in human diseases such as osteoporosis [11, 12] and hypertrophic cardiomyopathy [13].

Since 2016 our group has demonstrated a new role for *Klf10* in skeletal muscle [14], specifically, relating to mitochondrial function [15]. Interestingly, deletion of *Klf10* results in defects in oxidative and glycolytic muscles as a result of altered mitochondrial functions relating to defects in the respiratory chain and enzyme activities. In light of these findings, it was of interest to analyze other tissues where the density of mitochondria is known to be involved in the cerebellum which contributes to the coordination, accuracy and synchronization of movements [16, 17]. Although little information is reported on a possible role for *Klf10* in neuronal function, Alvarez-Rodriguez *et al*. [18] have shown that cessation of cell proliferation is regulated by *Klf10*, prior to differentiation of cerebellar granular neuron precursors. *Klf10* also belongs to a group of five genes identified as nerve growth factors [19] and has been linked to antiproliferative functions in the signaling pathway of PC12 cells [20]. Indeed, Wibrand *et al*. [21] suggested that *Klf10* might be a target gene of neurotrophins. Dijkmans *et al*. [19] indicated that *Klf10* mediates the development of a dividing and undifferentiated precursor cell into a differentiated neuron during neuronal development and neurogenesis. Furthermore, it has been shown that *Klf10* promotes cell cycle arrest in cerebellum granular neuron precursors (CGNPs).

For these reasons, we sought to use complementary techniques applied *in vivo* (magnetic resonance imaging and spectroscopy tests, behavior analysis) and *in vitro* (spectroscopy analysis, enzymes activities) to decipher the role of *Klf10* on the structural (histology, texture, diffusion) properties and the mitochondrial activities of the mouse cerebellum.

## 2. MATERIALS AND METHODS

### 2.1. Animals

The generation of *Klf10* KO mice has been previously described [22]. To be consistent with our previous studies performed only on female mice aged 3 months, we have also utilized the same type of 3 animals derived from heterozygous breedings. All mice were maintained in a temperature controlled room (22°C ± 2°C) with light/dark cycle of 12 hours. Animals had free access to water and were fed with standard laboratory chow *ad libitum*. The protocol was approved by the French ministry of higher education, research and innovation (Permit Number: DUO-4776), the local ethics committee Comité Régional d’Ethique en Matière d’Expérimentation Animale de Picardie (CREMEAP; Permit Number: APAFIS #8905–2021011109249708) and the local ethics committee Comité Régional d’Ethique en Matière d’Expérimentation Animale des Pays de La Loire (APAFIS #8186–2016121315485337) for behavior experiments.

### 2.2. Morphological Analysis

WT (N = 3) and *Klf10* KO (N = 3) mice were sacrificed by intracardiac perfusion of PFA (4% in PBS) followed by overnight post-fixation of the whole brain in a 4% PFA solution at 4°C. 80 μm thick sagittal sections were prepared using a Leica VT1000S vibratome. Slices were permeabilized for 30 minutes in Permeabilization Buffer (PB: 1x PBS supplemented with 0.1% Triton X100 and 1% BSA), then incubated overnight with primary antibodies diluted in PB: Rabbit anti-MAP2 (Millipore, 1:1000) and Mouse anti-Neurofilament (BioLegend, clone [SMI-312], 1:1000), or Mouse anti-NeuN (abcam, clone [1B7], 1:500) and Rabbit anti-Parvalbumin (abcam, 1:500). The following day, sections were washed 3 times in 1x PBS and subsequently incubated in secondary antibody-containing PB (goat anti-mouse antibody, Alexa 488, 1:2000, life technology, and goat anti-rabbit antibody, Alexa 546, 1:2000, life technology). Nuclear DNA was stained using Hoechst 33258 (1:5000). Confocal images were acquired in 1024 × 1024 mode with a Nikon Ti-E microscope equipped with the C2 laser scanning confocal microscope. We used the following objective lenses (Nikon): 10× PlanApo; NA 0.45, 20× PlanApo VC; NA 0.75. Microscope control and image analysis was performed using the Nikon software NIS-Elements (Nikon).

### 2.3. Magnetic Resonance Imaging (MRI)

**Figure 1.**
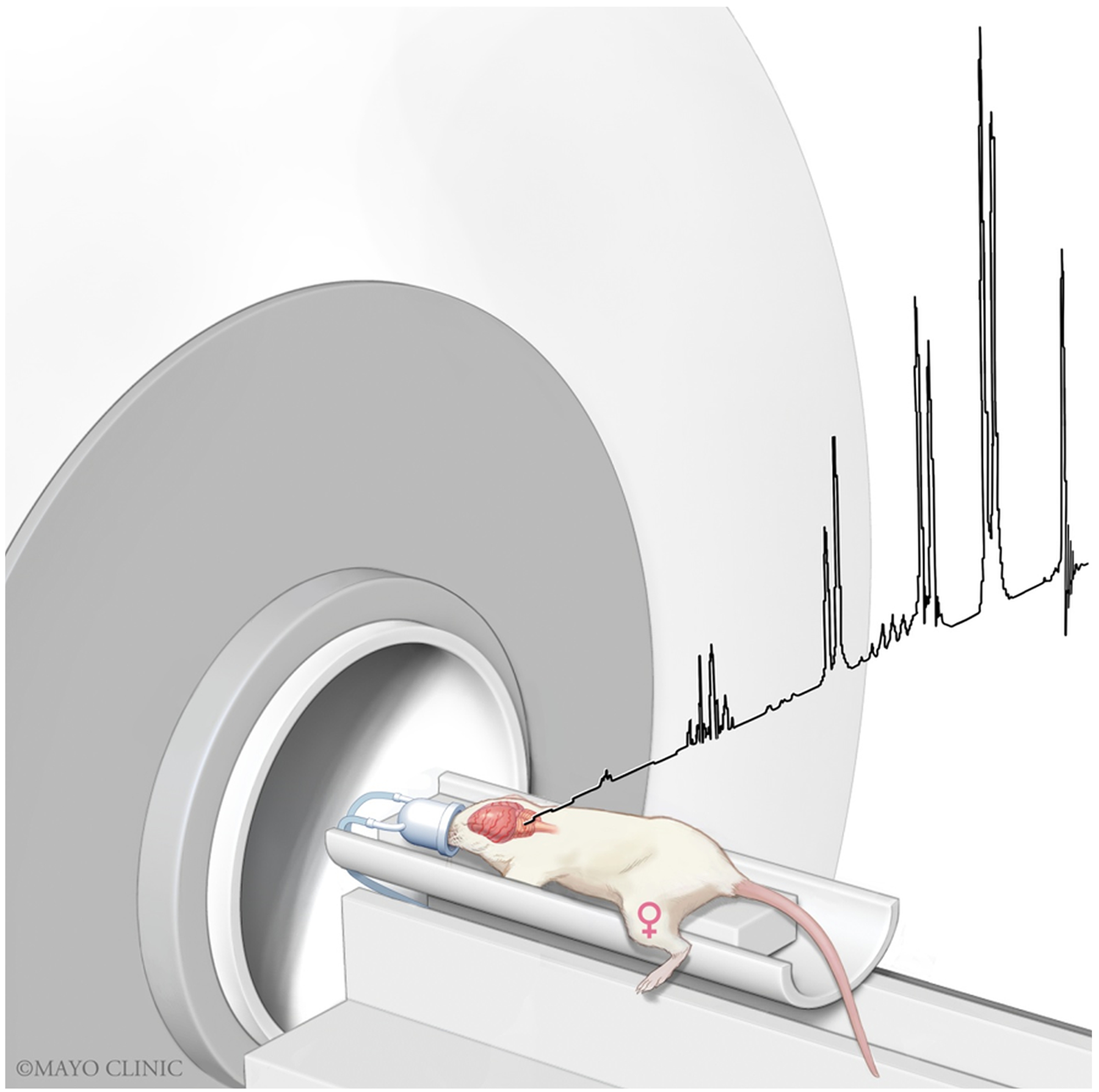
*In vivo* acquisition of the cerebellum (WT vs *Klf10* KO mice) using MRI machine (9.4T) to analyze the texture, the diffusion and to quantify the metabolites.

MRI acquisition was performed on a 9.4T horizontal ultra-shielded superconducting magnet dedicated to small-animal imaging (94/20 USR Bruker Biospec, Wissembourg, France) and equipped with a 950 mT/m gradient set (Figure 1). A birdcage coil (35 mm inner diameter) was used for both proton transmission and reception.

Mice were anesthetized during the MRI experiment with 1.5% isoflurane and a mixture of O2/air (1:1) at an output of 0.5 L/min. Respiration was monitored during the entire experiment, and body temperature was maintained at 37°C using a warm-water circulation system.

Axial and coronal images of the *Klf10* KO (N = 10) and wild-type (WT) (N = 9) cerebellum were obtained to be quantified with texture analysis using a gradient echo (Flash) sequence with the following parameters: TE/TR = 6 ms/252ms, flip angle = 20°, FOV size = 2 × 2 cm, matrix size = 256 × 256, bandwidth = 50 kHz, slice thickness = 570 μm, to display 78 × 78 μm^2^ in plane resolution for a duration of 1 min (one accumulation). A region of interest (ROI) was chosen in the cerebellum and texture parameters (grey levels, run length, …) were analyzed with correspondence factorial analysis using our previously reported protocol [23].

Subsequently, a spin echo diffusion-weighted sequence was applied to 10 mice (*Klf10* KO (N = 5) and WT (N = 5)) using the following parameters: TE/TR = 27/1500ms, flip angle = 90°, FOV size = 2.5 × 2.5 cm, matrix size = 128 × 128, b values = 8, 200 and 400 s/mm^2^ in x, y and z directions. Diffusion (D) coefficients Dx, Dy and Dz respectively in x, y and z direction were calculated with Bruker Paravision 5.1 software. They correspond to the slope of the decreasing line after the representation of ln(S/S_0_) = f(b), where S_0_ corresponds to the grey levels mean of the cerebellum ROI without diffusion and S the grey levels mean of the cerebellum ROI with diffusion.

### 2.4. *In Vivo* Magnetic Resonance Spectroscopy (MRS)

For MRS acquisition, static B0 homogeneity was first adjusted with first and second order shims in a cubic (3.5 × 3.5 × 3.5 mm) voxel centered in the cerebellum with the Bruker Fastmap procedure [24]. The half-height linewidth achieved for tissue water was less than 18 Hz. A PRESS (Point Resolved Spectroscopy) sequence was used to record localized ^1^H spectra in a (3 × 3 × 3 mm) voxel placed in fastmap cubic voxel with the following parameters (TR = 4 s, TE = 16 ms, 256 scan: 17 min, 2048 points, bandwidth = 4000 Hz) with water suppression using VAPOR (VAriable Pulse power and Optimized Relaxation delays) module and outer volume suppression [25]. Eddy current compensation and static magnetic field drift correction were applied during spectra acquisition.

MRS Spectra were analyzed with a time-domain method described in Ratiney *et al*. 2010 [26], using the CSIAPO software developed by Dr Le Fur (CRMBM, Marseille, France) and Dr Ratiney (CREATIS—INSA, Lyon, France) which is a spectral analysis tool for quantification algorithm QUEST (quantification based on quantum estimation).

QUEST is a linear combination model decomposing free induction decay (FID) signal in temporal domain. QUEST uses a database to fit a weighed combination of metabolite signals and then to obtain a quantification in arbitrary units (A.U.). This database is simulated through GAVA (GAmma Visual Analysis) which is used to model metabolite signals including their characteristics (chemical shift, amplitude and phase) and also experimental parameters (magnetic field and echo time).

The following cerebellum metabolites were then quantified: choline (Cho), creatine (Cre), glutamine (Gln), glutamate (Glu), myo-inositol (Myo), N acetyl aspartate (Naa) and taurine (Tau).

### 2.5. *In Vitro* ^1^H-NMR Spectroscopy

Prior to NMR analysis, the dried cerebellum samples (N_WT_ = 5 and N_*Klf10* KO_ = 5) were reconstituted in 220 μL of deuterated buffer containing TSP (3-trimethylsilylpropionic acid) at 145 μmol·L^−1^ and transferred to conventional 3 mm NMR tubes (CortecNet). As previously described, the extraction protocol of metabolites from cerebellum was adapted from the Folch type two-step procedure described by Wu *et al*. [27]. ^1^H-NMR spectra were obtained with a Bruker DRX-600 AVANCE-III HD spectrometer (Bruker SADIS, Wissembourg, France), operating at 14 T, with a TCI cryoprobe. Standard water suppressed ^1^H-NMR spectra were acquired at 298 K using a “noesypr1d” pulse sequence with relaxation delay of 20 s. Spectra were processed using Topspin version 3.2 software (Bruker Daltonik, Karlsruhe, Germany). As previously described [28], ^1^H-NMR spectra were automatically reduced to ASCII files using AMIX Software package (Analysis of MIXture, version 3.9.14, Bruker Biospin, Karlsruhe, Germany). Spectral intensities were scaled to the total spectral intensity, and the resulting data table was analyzed by multivariate and univariate statistical analyses.

### 2.6. Statistical Analysis

For multivariate analysis, Partial Least Square Discriminant Analysis (PLS-DA) was performed using SIMCA-P+ Software (version 13.0, Umetrics, Umeå, Sweden) on the NMR dataset containing WT (N = 5) and *Klf10* KO (N = 5) cerebellum values. All data sets were scaled to unit of variance allowing all metabolites to become equally important. The PLS-DA is a classification and prediction method that allows the identification of spectral features (metabolites) that define the separation between experimental groups (phenotypes). Variable Importance in the Projection (VIP) > 1 were considered as the most contributive in the phenotype separation.

For univariate analysis, Student’s t-test was performed using MetaboAnalyst [29] for all variable importance on projection (VIP). A p-value < 0.05 was considered as significant.

Heatmaps were generated to allow for visualization of data. Each colored cell on the map corresponds to an intensity value in our dataset. Considering the VIP > 1 and a p-value < 0.05, a heatmap was displayed for the cerebellum.

### 2.7. Mitochondrial Enzymes Activities

The enzyme activities of citrate synthase (CS), mitochondrial NADH: coenzyme Q oxidoreductase (Complex I) and cytochrome oxidase (COX, Complex IV) were evaluated. Briefly, cerebellum tissues (N_WT_ = 6, and N_*Klf10* KO_ = 6) were harvested and rapidly frozen in azote solution before storage at −80°C. Subsequently, the extraction of enzymes were performed with an ice-cold buffer (50 mg·ml^−1^; containing (in mM): HEPES 5 (pH 8.7), EGTA 1, dithiothreitol 1, and 0.1% Triton X-100) using a Bertin Precellys 24 homogenizer (Bertin, Montigny-le-Bretonneux, France) and the enzyme activities were measured using standard spectrophotometric protocols [30, 31].

### 2.8. Behavior Experiments

The *in vivo* tests were blindly performed in the same sequence for each mouse, with equivalent time of rests in between, and at the same time of the day. Mice were randomly assigned to 2 groups according to genotype *Klf10* KO (N = 10) and WT (N = 7) as described above.

#### 2.8.1. Clasping Reflex and Grip Test

The hindleg clasping reflex assesses the inhibitory function of the central nervous system while a mice is suspended by its tail [32]. Mice with neurological impairment show abnormal reflex retraction of the legs and paws. A score of zero corresponded to normal placement, one to inconsistent retraction of one leg, two to permanent retraction of one leg, three to inconsistent retraction of both legs and four to permanent retraction of both legs.

The skeletal muscle force was assessed by using the grip test. Mice were placed with their four paws on a grid and were pulled backward until they released their grip. The measured forces of the four limb were expressed in absolute values (g) or values related to the mouse body weight (g/g BW). The peak force generated was measured with a grip meter (Bio-GT3, Bioseb, France), as described before by Carré-Pierrat *et al*. [33]. The mean result of three assays was normalized to the body weight.

#### 2.8.2. Actimeter Test

The locomotor behavior was examined with an open field actimeter [34]. For this analysis, mice were individually placed in an automated photocell activity chamber (Letica model LE 8811, Bioseb, France) which consists of a plexiglass chamber (20 × 24 × 14 cm) surrounded by two rows of infrared photobeams. The first row of sensors was raised at a height of 2 cm for measuring horizontal activity and the second row placed above the animal for vertical activity. The spontaneous motor activity was measured for 5 min using a movement analysis system (Bioseb, France), which dissociates activity time (s), distance traveled (cm), stereotyped and number (nb) of reaching movements.

## 3. RESULTS

### 3.1. Morphological Analysis

**Figure 2.**
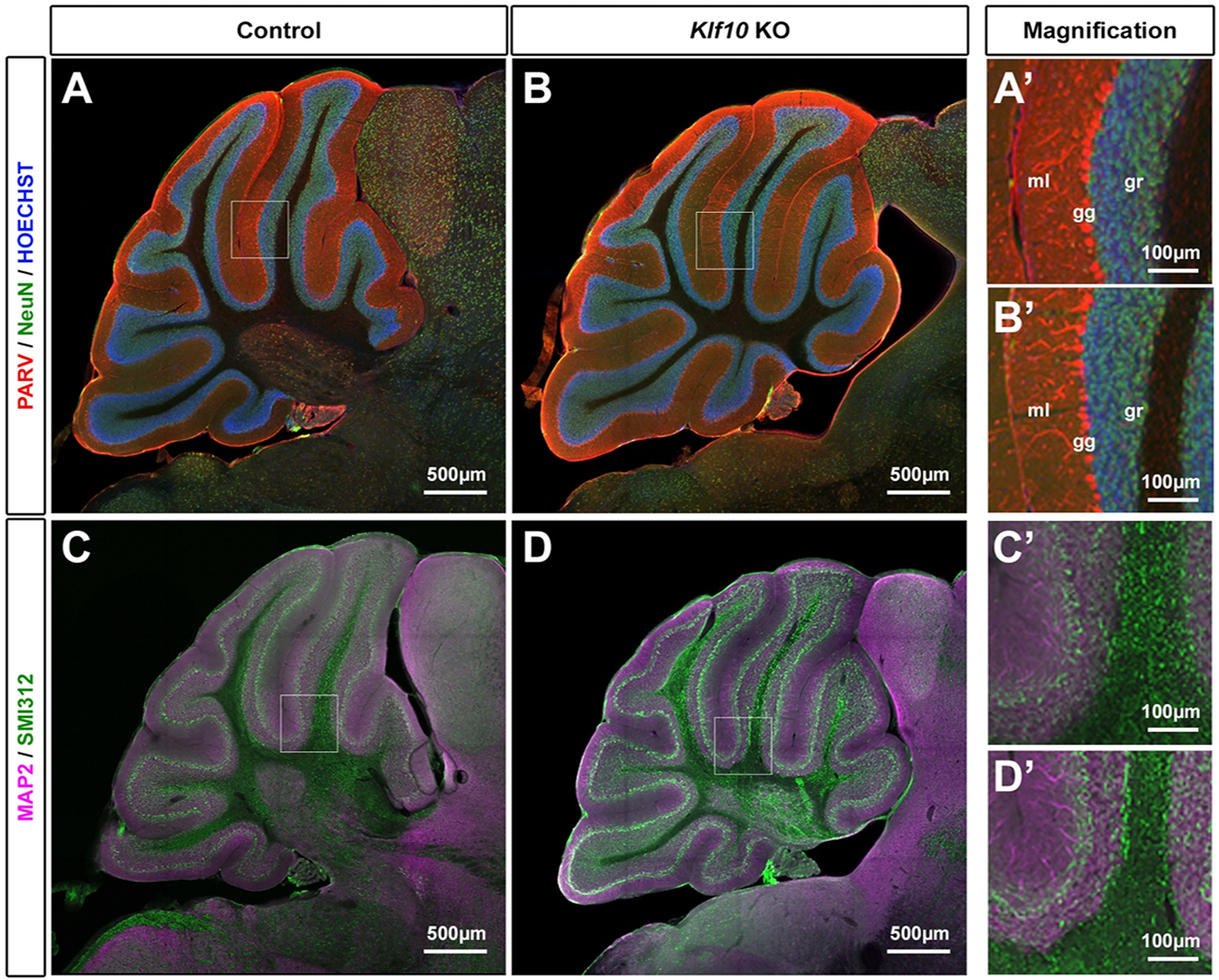
Histological analyses of the cerebellum of *Klf10* KO mice compared to wild-type littermates. (A)-(B): Overall structure of the cerebellum with the magnification in (A’)-(B’). ml: molecular layer. gg: ganglionic layer (Purkinje cell). gr: granular layer. (C)-(D): Repartition of the dendritic (MAP2) and axonal (SMI312) with the magnification in (C’)-(D’).

At first, we performed histological analyses of mouse brains (Figure 2). There was no change overall of the size and structure of the cerebellum, as the different lobules could be identified. We marked cerebellar neurons with the pan-neuronal marker NeuN, as well as the Purkinje-neurons marker Parvalbumin (Figure 2(A), Figure 2(B)). Overall we saw no change in the granular (gr), ganglionic (gg) and molecular (ml) layers organization in *Klf10* KO mice compared to control littermates (Figure 2(A’), Figure 2(B’)). Layers appeared similar in size and a single layer of Purkinje neurons was clearly visible. In parallel, dendrites (MAP2) and axons (neurofilaments: SMI312) were stained. Confocal analyses revealed a normal segregation of the somatodendritic and axonal compartments (Figure 2(C), Figure 2(D), magnification in Figure 2(C’), Figure 2(D’)). Collectively, our results suggest that the global architecture of the cerebellum is normal in *Klf10* KO animals. A future direction of the morphological analysis would be to examine the cellular level of the structure using higher resolution confocal microscopy or electron microscopy.

### 3.2. Texture Analysis

**Figure 3.**
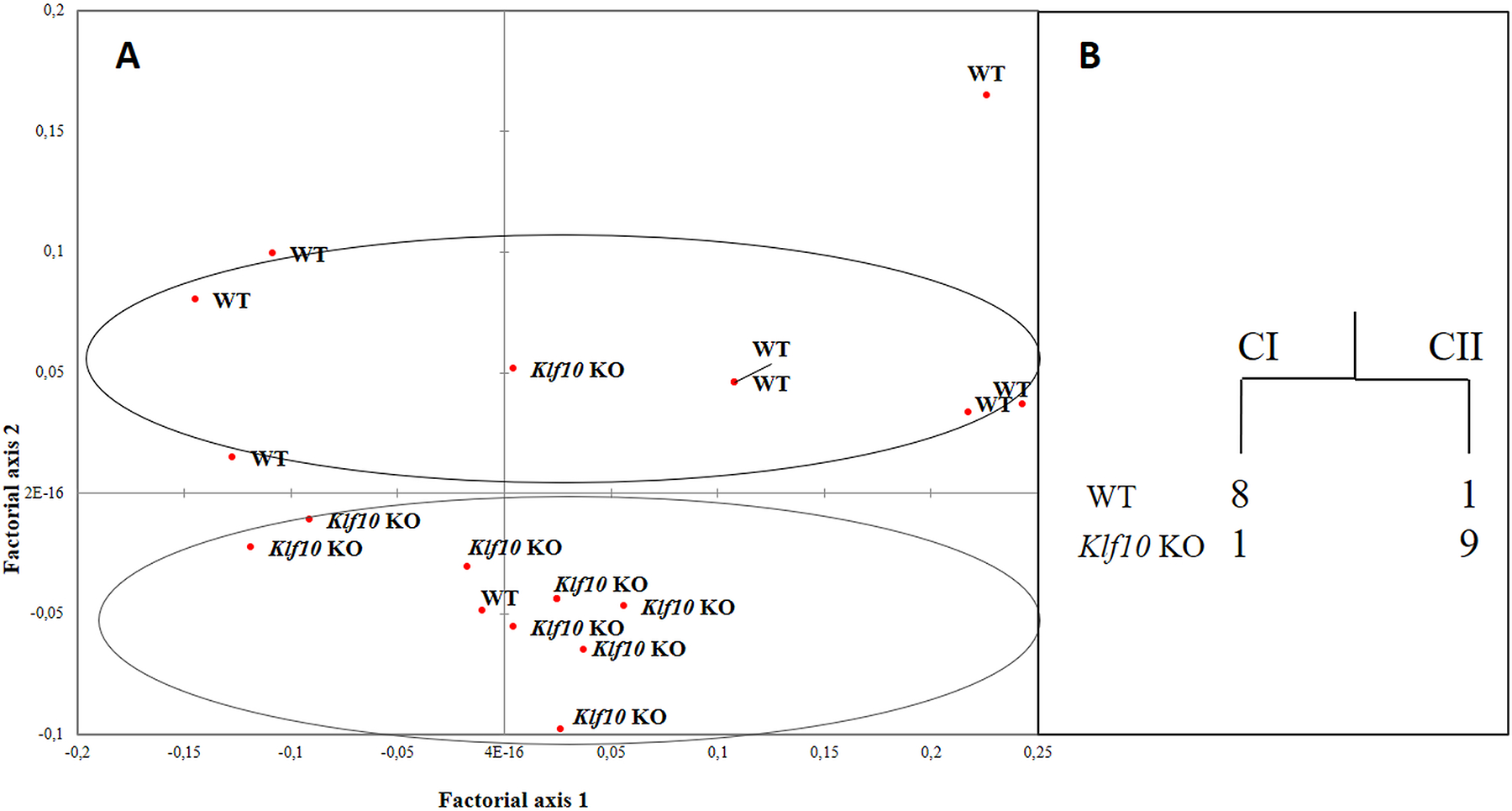
(A) Correspondence factorial analysis and (B) hierarchical ascending classification of cerebellum in function of genotype (WT vs *Klf10* KO). WT: wild type; *Klf10* KO: knockout Krüppel-like factor 10. CI: class I; CII: class II.

Figure 3 illustrates the results of the multiparametric analyses of the cerebellum texture profiles obtained for WT and *Klf10* KO mice from correspondence factorial analyses (CFA) and dendrogram representations. Both results reveal 2 distinct groups of texture profiles as a function of mouse genotype. Hierarchical ascending classifications (HAC) reveal that class I (CI) has a majority of WT cerebellum (N = 8) compared with class II (CII), which has the most *Klf10* KO cerebellum (N = 9). The most discriminating (p < 0.05) texture parameters were mean, run length distribution (0°, 45°, 90°, 135°) and grey level distribution (0°, 45°, 90°, 135°). These results demonstrate the differing texture profiles between WT and *Klf10* KO cerebellum. The global values for the cerebellum texture analysis were found to be 89% (WT vs. *Klf10* KO).

### 3.3. Diffusion

**Figure 4.**
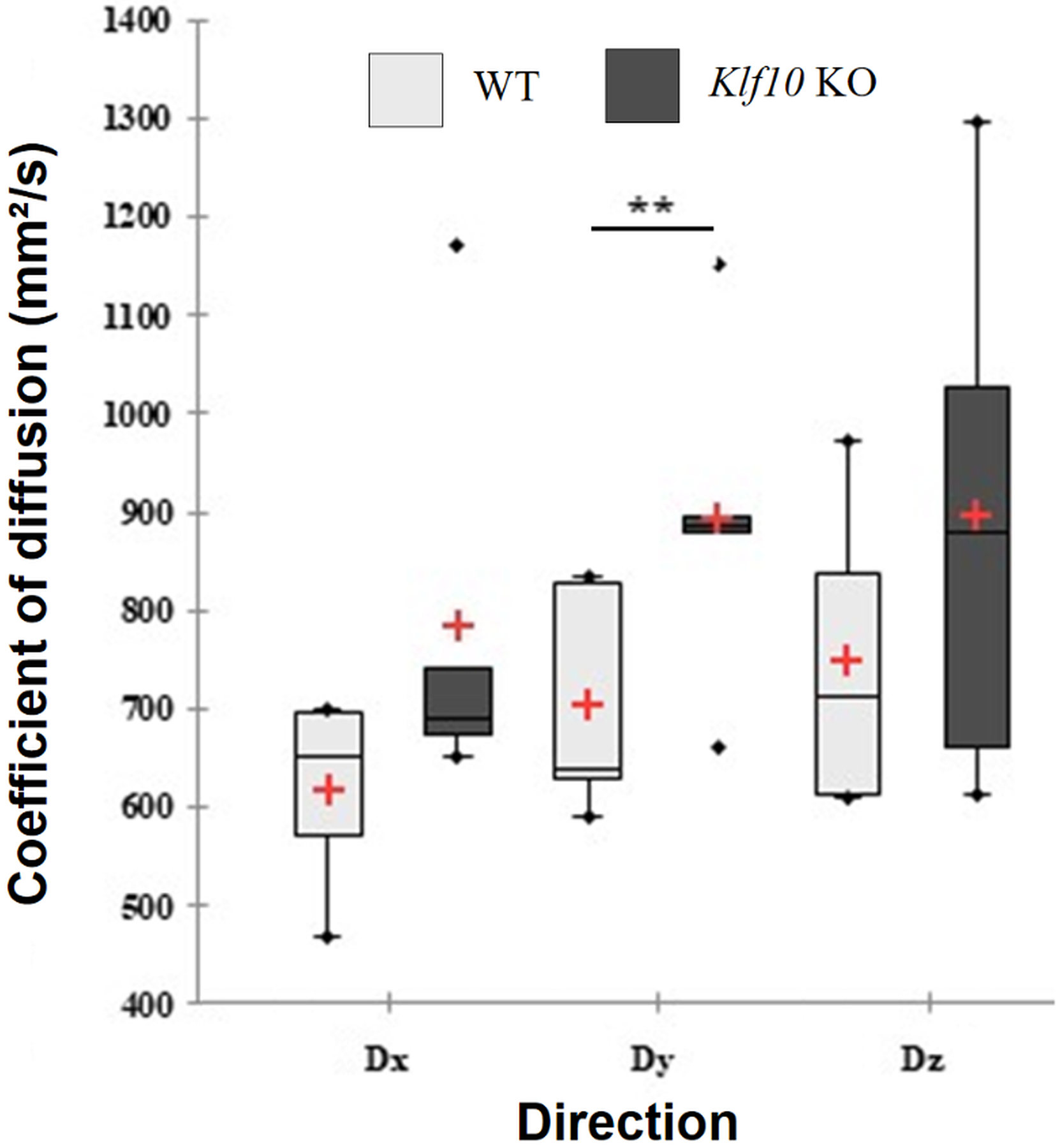
Coefficient of diffusion (mm^2^/s) for the cerebellum. The ends of the lower and upper whiskers of each boxplot indicate the minimum value and the maximum value respectively. Median and mean values are represented respectively with a black line and a red cross. Dx, Dy, Dz: diffusion coefficients in x, y and z directions. *Klf10* KO: knockout Krüppel-like factor 10. WT: wild type. **: P < 0.01.

The diffusion coefficients measured within the WT and KO cerebellum for each direction are illustrated in Figure 4. Only the coefficient of diffusion (Dy), calculated in y direction, was significantly (p < 0.01) different as a function of genotype. The extreme, the mean (red cross), and the median (solid line in the box) values of Dx, Dy and Dz were plotted as a function of mouse genotype.

### 3.4. Metabolites Quantification

**Figure 5.**
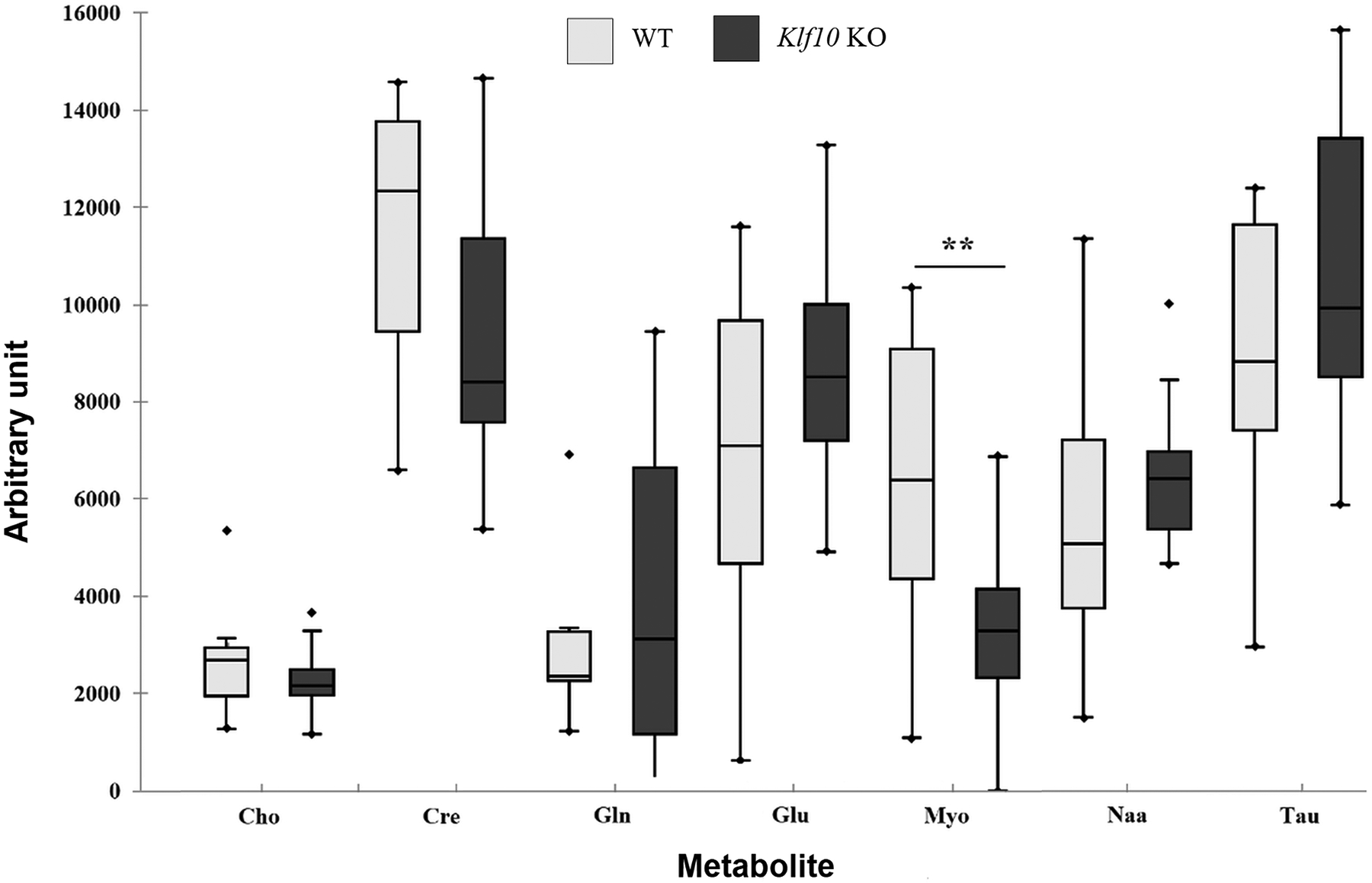
Box-whisker plots of cerebellum metabolites obtained for WT and *Klf10* KO mice using *in vivo* ^1^H-MRS. Cho: choline. Cre: creatine. Gln: glutamine. Glu: glutamate. *Klf10 KO*: knockout Krüppel-like factor 10. Myo: myo-inositol. Naa: N acetyl aspartate. Tau: taurine. WT: wild type. **: P < 0.01.

*In vivo* acquisition of the cerebellum metabolite is shown in Figure 5 with box plots representing the extreme, the mean (red cross), and the median (solid line in the box) values of the 7 metabolites as a function of mouse genotype. The amount of myo-inositol decreased significantly (p = 0.009) in *Klf10* KO cerebellum compared to the WT mice. The remaining metabolites (choline, creatine, glutamine, glutamate, N acetyl aspartate, taurine) showed no significant difference between the WT and KO mouse cerebellum.

### 3.5. *In Vitro* ^1^H-NMR Spectroscopy

**Figure 6.**
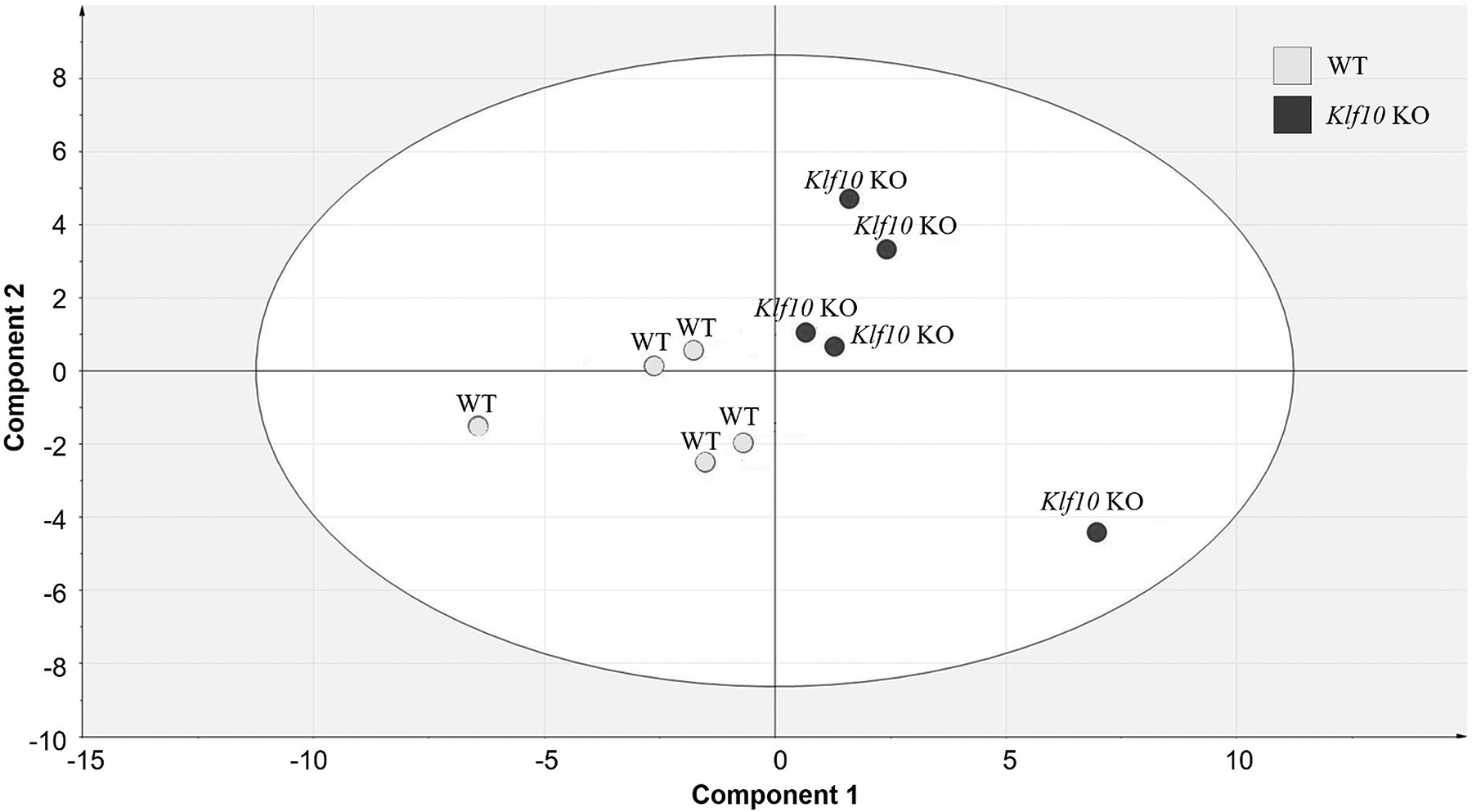
Partial Least Square Discriminant Analysis (PLS-DA) score resulting from modeling the ^1^H-NMR spectral data matrix of WT (N = 5) and KO (N = 5) cerebellum, R^2^Y (cum) = 0.76, Q^2^ = 0.7, X = 25. *Klf10* KO: knockout Krüppel-like factor 10. WT: wild type.

The PLS-DA model obtained from the NMR dataset containing the WT and *Klf10* KO cerebellum (Figure 6) revealed a significant separation as a function of mouse genotype (predictive ability Q^2^ = 0.7, goodness of fit R^2^Y (cum) = 0.76). The PLS-DA distribution confirms differences between WT and KO metabolic profiles.

**Figure 7.**
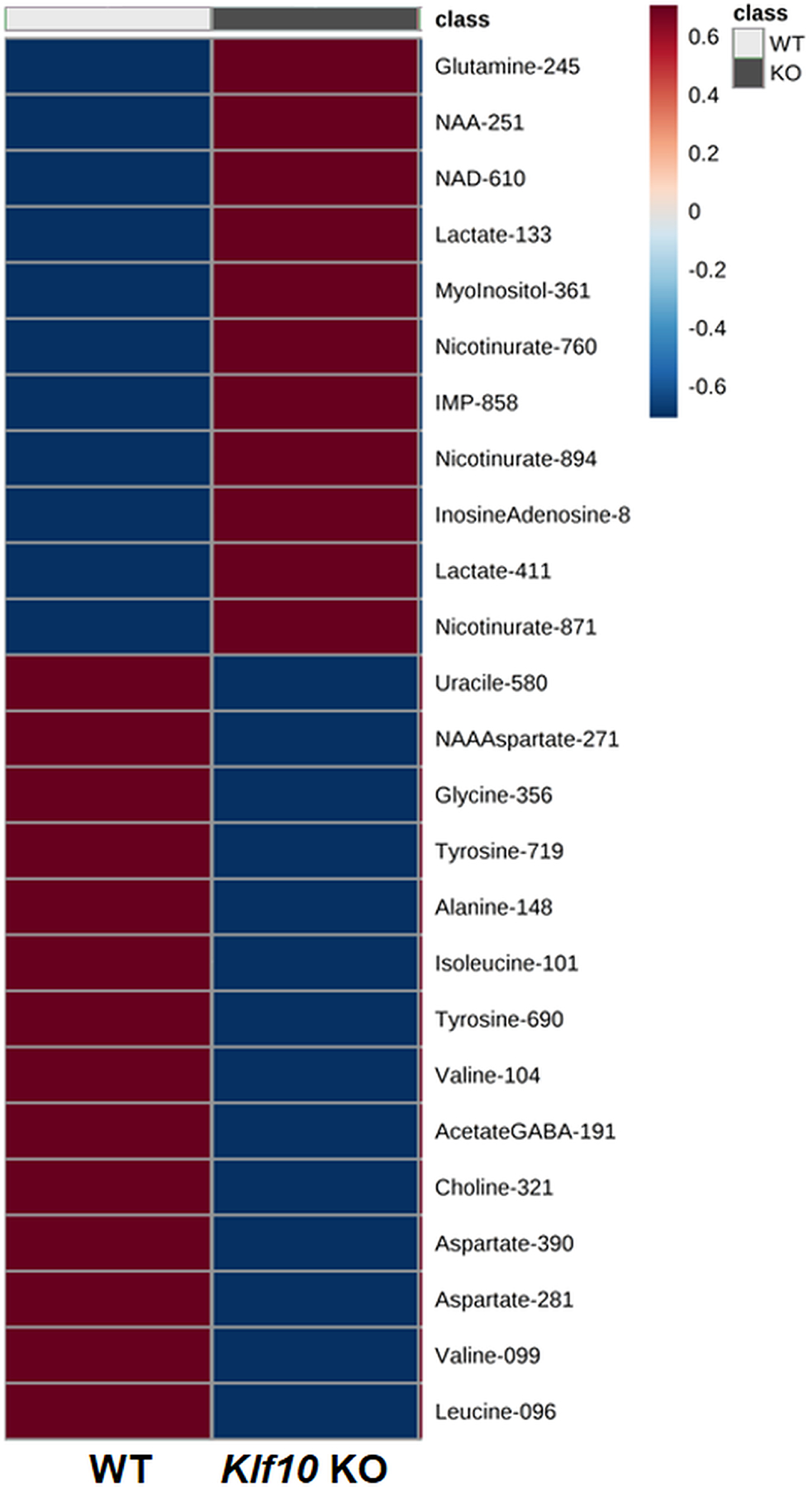
The heatmap displays the top 25 features ranked by PLS-DA to retain the most contrasting patterns. The heatmap shows only group averages.

The PLS-DA permitted the clustering of WT vs KO using 25 variables. Figure 7 depicts a heatmap generated with the top 25 features ranked by the PLS-DA models. Over these 25 variables, the PLS-DA model allows for the identification of 9 variables of importance in the projection (VIP > 1) demonstrating the biological relevant changes in the metabolome of the compared groups (Table 1). Deletion of *Klf10* induced a significant up regulation of two metabolites (lactate, nicotinurate) with a trend for inosine + adenosine region and NAD+, and the significant down regulation of five metabolites (tyrosine, valine, isoleucine, alanine) with a trend for NAA + aspartate region.

### 3.6. Mitochondrial Activity

**Figure 8.**
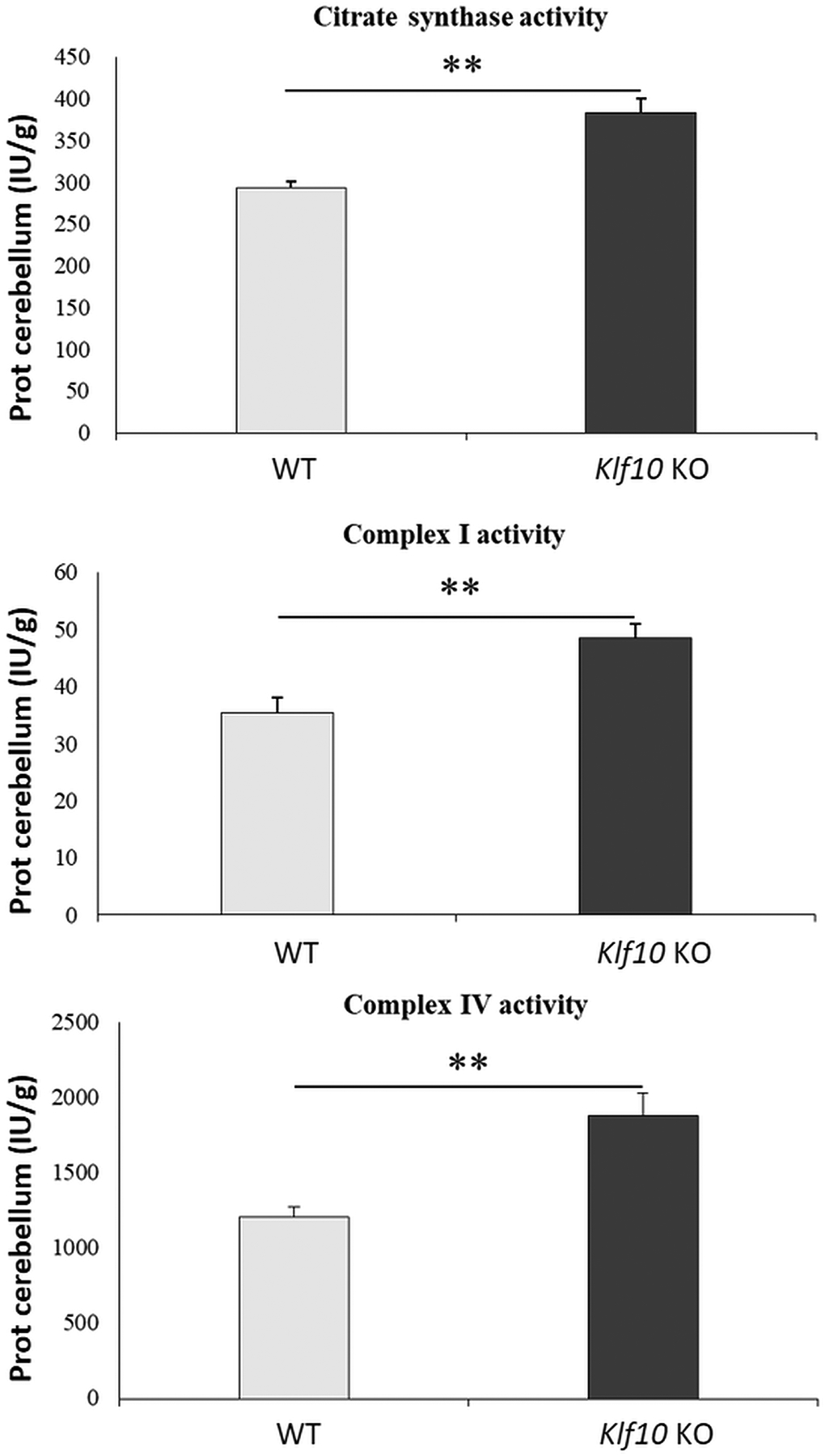
Mitochondrial enzyme activities in the cerebellum of WT (wild type) and *Klf10* KO (knockout Krüppel-like factor 10) mice. Activities were reported as μmol·min^−1^·g^−1^ protein (μmol·min^−1^, international units, IU). **: P < 0.01.

Significant increase (P < 0.01) in the enzymatic activities for the citrate synthase (CS), the NADH: ubiquinone oxidoreductase (Complex I) and the cytochrome oxidase complex (COX, Complex IV) were detected in *Klf10* KO cerebellum compared to WT cerebellum (Figure 8).

### 3.7. Behavior Experiments

**Figure 9.**
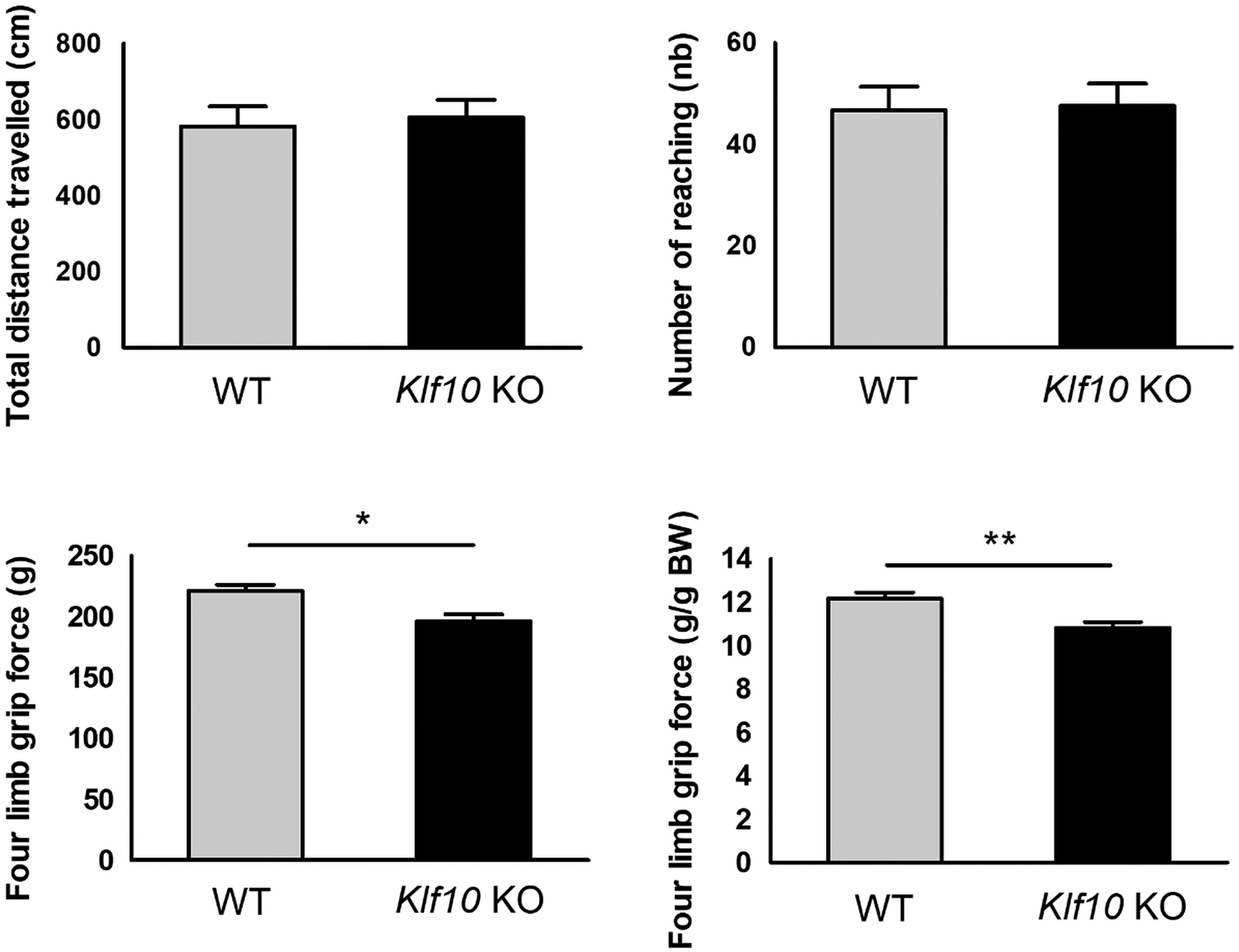
Actimeter and grip test parameters from WT (N = 7) and *Klf10* KO (N = 10) mice. *: P < 0.05, **: P < 0.01. *Klf10* KO: knockout Krüppel-like factor 10. WT: wild type.

We have observed that clasping reflex experiments showed no significant difference between *Klf10* KO (N = 10) and WT (N = 7) mice and the score equivalent to zero showed normal reflex retraction of the legs and paws. These results are in good agreement with the analysis of the spontaneous locomotion that showed no significant difference between the WT and *Klf10* KO mice (Figure 9). With regard to these measures, there is no apparent effect on behavior in *Klf10* deficient animals. An interesting result was observed using the grip test measurements. As shown in Figure 9, *Klf10* KO mice showed lower grip strengths compared to WT mice.

## 4. DISCUSSION

We demonstrated that deletion of *Klf10* results in a significantly faster diffusion of water molecules in *Klf10* KO cerebellum along the main direction (y), which corresponds to the displacement of the molecules perpendicularly to the axis of the fiber tissue. This interesting result could indicate a loss of interfaces between the different layers of the tissue or a loss of cell density in *Klf10* KO cerebellum. This physiological change is coherent with the texture result which confirmed at the level of the pixel the changes in composition of the cerebellum tissue. Indeed, run lengths were selected as one of the most discriminating texture parameters. A run corresponds to pixels that have the same grey levels (*i.e.* a homogeneous area). Thus, the loss of interface demonstrates that there are longer run lengths.

In addition, the significant decrease of the myo-inositol (Myo) inside the *Klf10* KO cerebellum compared to WT littermates, as obtained via *in vivo* MRS acquisition, is in agreement with the increase of diffusion found in the KO mice. Indeed, a decrease in Myo metabolite indicates an osmotic problem [35–37] and this phenomenon is often observed in hepatic encephalopathy, which corresponds to brain damage that occurs when the liver no longer functions properly [38]. While cirrhosis has not been observed in *Klf10* KO mice under standard conditions, it should be noted that hepatic expression of *Klf10* is increased in fatty liver of obese mice [39] and involved in several mechanisms which are dysregulated in NAFLD (Non-alcoholic fatty liver diseases) [40–42].

Interestingly, the myo-inositol as measured *in vitro* from crushed cerebellum samples using ^1^H-NMR spectroscopy, was found to be up regulated in *Klf10* KO tissue while a down regulation was observed *in vivo* using MRS acquisition. These opposing results could be due to the different environments (*in vivo* vs *in vitro*) of the tissues.

The deletion of *Klf10* induced changes in mitochondrial activity as reflected by a significant increase in the enzyme activities of the respiratory chain and that of citrate synthase. These results are consistent with the up regulation of NAD^+^ and glutamine which attempt to compensate the perturbation of the Krebs cycle. Based on these results, it is of interest to further elucidate the mechanisms by which *Klf10* modulates mitochondrial function.

In the cytosol, the up regulation of lactate in *Klf10* KO cerebellum demonstrates an increased source of energy, which could be explained by a dysregulation of the mitochondria through an increase in enzyme activities. Interestingly, lactate was also up regulated in fast twitch skeletal muscle [15] of *Klf10* KO mice while the mitochondrial activity was decreased. Thus, there is no parallel between the impact of *Klf10* depletion on mitochondrial metabolism in muscle and cerebellum. However, the present results further implicate *Klf10* as a critical regulator of mitochondrial activity and energy production. Additional defects in *Klf10* KO mitochondrial activity were also revealed by the down regulation of the amino acids which belong to the family of the glucoformer (alanine, valine, glutamine, glycine) or both glucoformers and ketoformers (isoleucine, tyrosine) or exclusively ketoformers (leucine). Interestingly, these amino acids are implicated in different metabolic pathways (gluconeogenesis and ketogenesis), providing additional evidence for the role of *Klf10* in energy production. These results are coherent with the studies implicating *Klf10* as a regulator of hepatic glucose in mice [43] and its involvement in hepatic gluconeogenesis through the direct transcriptional repression of Pepck [10].

Moreover, the up regulation of NAA (N-Acetyl-Aspartate) indicates an increase in the activity of neuronal state [44] which could be explained by the important consumption of energy. This metabolite is a marker of neuronal density, function, and future studies will aim to further investigate the cellular properties of the neuronal cells. Interestingly, the *Klf10* KO mice did not show a different behavior when compared with their normal littermate controls. The decrease in the force for the *Klf10* KO mice is coherent with the recent study demonstrating *Klf10* as a regulator of the contractile properties of skeletal muscle fibers [45]. However, it is unclear if this effect is originated from alterations within the brain or is caused by the known defects in skeletal muscle of *Klf10* KO mice. In addition, more focused tests could be conducted, such as footprinting and balance beam tests, to further analyze the cerebellar motor deficits.

## 5. CONCLUSION

We hypothesize that *Klf10* is an important regulator of energy metabolism in mitochondria in cerebellum and skeletal muscle. However, the mechanisms by which *Klf10* regulates these processes in these tissues have yet to be elucidated.

## Figures and Tables

**Table 1.** Metabolites allowing the discrimination on the PLS-DA model between WT and *Klf10* KO cerebellum tissue. The variables of importance on the projection (VIP), the p-values obtained with student t-test and the fold-change (FC) are expressed in the table. The up or down regulation in *Klf10* KO/WT is indicated.

Primary metabolite	VIP	FC	p-value	*Klf10* KO/WT
**Lactate**	1.360	1.16	0.006	Up
**Inosine + Adenosine**	1.291	1.28	0.080	Up
**Nicotinurate**	1.206	1.16	0.018	Up
**NAD** ^**+**^	1.130	1.25	NS (0.20)	Up
**Tyrosine**	1.115	0.63	0.034	Down
**Valine**	1.100	0.60	0.037	Down
**Isoleucine**	1.097	0.60	0.038	Down
**Alanine**	1.083	0.70	0.044	Down
**NAA + Aspartate**	1.047	0.94	NS (0.11)	Down

NAD: Nicotinamide adenine dinucleotide. NAA: N-Acetylaspartic acid. NS: No significant.
